# Mixed Neuropathologies, Neural Motor Resilience and Target Discovery for Therapies of Late-Life Motor Impairment

**DOI:** 10.3389/fnhum.2022.853330

**Published:** 2022-03-24

**Authors:** Aron S. Buchman, David A. Bennett

**Affiliations:** ^1^Rush Alzheimer’s Disease Center, Rush University Medical Center, Chicago, IL, United States; ^2^Department of Neurological Sciences, Rush University Medical Center, Chicago, IL, United States

**Keywords:** motor decline, neuropathology, resilience, aging, genomics

## Abstract

By age 85, most adults manifest some degree of motor impairment. However, in most individuals a specific etiology for motor decline and treatment to modify its inexorable progression cannot be identified. Recent clinical-pathologic studies provide evidence that mixed-brain pathologies are commonly associated with late-life motor impairment. Yet, while nearly all older adults show some degree of accumulation of Alzheimer’s disease and related dementias (ADRD) pathologies, the extent to which these pathologies contribute to motor decline varies widely from person to person. Slower or faster than expected motor decline in the presence of brain injury and/or pathology has been conceptualized as more or less “resilience” relative to the average person This suggests that other factors, such as lifestyles or other neurobiologic indices may offset or exacerbate the negative effects of pathologies *via* other molecular pathways. The mechanisms underlying neural motor resilience are just beginning to be illuminated. Unlike its cousin, cognitive resilience which is restricted to neural mechanisms above the neck, the motor system extends the total length of the CNS and beyond the CNS to reach muscle and musculoskeletal structures, all of which are crucial for motor function. Building on prior work, we propose that by isolating motor decline unrelated to neuropathologies and degeneration, investigators can identify genes and proteins that may provide neural motor resilience. Elucidating these molecular mechanisms will advance our understanding of the heterogeneity of late-life motor impairment. This approach will also provide high value therapeutic targets for drug discovery of therapies that may offset the negative motor consequences of CNS pathologies that are currently untreatable.

## Late-Life Motor Impairment Is Common and Usually Untreatable in Aging Adults

By age 85, most older adults show some degree of motor impairment. The prevalence of motor impairment will vary with how motor function is assessed and the threshold employed for its impairment (Louis and Bennett, 2007; Rosso et al., 2013; Buchman et al., 2016a,b). Yet, a simple thought experiment suggests that progressive loss of motor function is ubiquitous; no one 85 years old can walk or run at the same speed or lift the same load achieved when they were 25 years old. While loss of motor function may be a typical accompaniment of aging, it is not benign, as it is associated with an increased risk of adverse health outcomes including different disabilities, impaired cognition i.e., MCI or dementia, loss of independence or institutionalization and mortality (Fried et al., 2004; Buchman et al., 2011, 2020a,2021a; von Coelln et al., 2019; Beeri et al., 2021).

Although illnesses such as broken hip, myocardial infarction, or stroke can cause acute motor impairment, more commonly loss of motor function develops gradually and imperceptibility. Consider for example declining walking speed and balance that occur in many older adults in the absence of overt disability or clinical diseases. Treatable neural causes of late-life motor impairment such as hydrocephalus, Parkinson’s disease or polymyositis are well-recognized but rare and account for only a small number of older adults (Askanas and Engel, 2002; Shulman et al., 2011). Thus, in most individuals a specific etiology and treatment to modify the inexorable progression of motor decline cannot be identified. The magnitude of the personal and societal costs of late-life motor impairment underscores the public health priority in filling knowledge gaps about its underlying mechanisms so that personalized medicine and targeted therapies can be developed.

### The Clinical Manifestations of Motor Decline Are Heterogeneous

Motor function is a complex behavior that depends on the orchestration of diverse non-CNS physiologic systems including cardiopulmonary, musculoskeletal, metabolism and motor control systems in the central nervous system (Ferrucci et al., 2000; Rosso et al., 2013). Neural control is crucial for motor planning, initiation and execution of all motor function. The neural systems subserving motor control are located in multiple interconnected cortical and subcortical regions which interact with basal ganglia and cerebellum (Jeannerod et al., 1995; Rizzolatti and Luppino, 2001; Fogassi and Luppino, 2005; Fogassi et al., 2005; Halsband and Lange, 2006; Lehericy et al., 2006). Descending white matter tracts provide the means for these supraspinal motor systems to influence motor systems in the spinal cord that *via* peripheral nerve directly regulate muscle firing, the final effector of all movement (Burke, 1981, 2002; Bizzi et al., 2002; Poppele and Bosco, 2003; Lanuza et al., 2004; Gordon and Whelan, 2006; Gosgnach et al., 2006).

Neural motor control is not a unitary process, but rather varied motor abilities derive from the coordinated activity of different subnetworks of the widely distributed motor control systems. These systems are dissociable, spatially distinct and extend from the brain to musculoskeletal elements in the periphery (Burke, 1981, 2002; Hikosaka et al., 2002). Damage or age-related degenerative changes within these widely distributed motor pathways can lead to strikingly heterogeneous motor manifestations. For example, many older adults commonly show reduced muscle strength and bulk, slowed gait speed and poor balance. These impairments can occur alone or in different combinations with a broad spectrum of severity due to the different sites and varied extent of damage within these widely distributed motor systems and also contributes to identifying treatments. Yet, even individuals with identical damage and clinical deficits at one point in time can show divergent trajectories of change over time with one individual showing minimal change and a second showing rapid decline. These observations highlight the importance of identifying the pathologic bases and molecular mechanisms that may account for the phenotypic heterogeneity late-life motor impairment as therapeutic interventions may vary for different underlying causes.

Identifying groups of older adults at increased risk for specific adverse health outcomes is crucial for efforts to develop and deploy early targeted treatments that can prevent progressive heterogeneous motor deficits and their consequences. To identify at risk adults, investigators have reported the usefulness of varied syndromes based on motor performances such as gait speed or grip strength alone or in combination with other clinical covariates to identify older adults at risk for adverse health outcomes. For instance, in the geriatric literature, sarcopenia is based on muscle strength and bulk (Baumgartner et al., 1998); physical frailty includes based on grip strength and gait speed; (Fried et al., 2001), while in the neurologic literature, parkinsonian signs are based on signs of bradykinesia, tremor, rigidity and parkinsonian gait (Louis et al., 2005); and various summary motor measures are based on testing a wider range of common motor performances (Onder et al., 2005; Buchman et al., 2007b). Previous studies have linked these different measures to all-cause mortality, (Bennett et al., 1996; Buchman et al., 2009) incident disabilities (Onder et al., 2005; Louis et al., 2006; Delmonico et al., 2007), mild cognitive impairment and dementia (Louis et al., 2005; Buchman et al., 2007a).

These various syndromes that have been tested may capture distinct aspects of late-life motor impairment that predict adverse health outcomes. Yet, there are few studies which modeled these syndromes together to determine to what extent they provide improved model performance for predicting adverse health outcomes (Buchman et al., 2011, 2021a). Moreover, several recent studies suggest that despite the use of different syndromic labels, motor function rather than non-motor covariates may be the primary drivers for their reported associations with adverse health outcomes (Cruz-Jentoft et al., 2019; Bhasin et al., 2020; Cawthon et al., 2020; Cesari and Kuchel, 2020; Patel et al., 2020; Beeri et al., 2021; Buchman et al., 2021a). Yet, even if these phenotypes predict adverse health outcomes, it is unclear that these different phenotypes alone or together predict distinct health outcomes.

For example, gait speed can be used to monitor overall health or rehabilitation efforts (Aoyagi et al., 2021) and is a well-known robust but non-specific predictor of risk of death, disabilities and cognitive impairment (Abellan van Kan et al., 2009). The complex distributed motor pathway which underlies gait speed may be affected by varied etiologies and may account for its sensitivity for manifesting dysfunction. Gait speed and poor motor function precedes and predicts incident cognitive impairment in many older adults (Yu et al., 2019a). Yet, testing gait speed alone cannot differentiate an individual who will develop cognitive impairment from one who will develop mobility disability. Similarly, sarcopenia, physical frailty, parkinsonism, motoric cognitive risk syndrome and composite motor measures have been reported to be associated with a wide range of adverse outcomes (Bennett et al., 1996; Louis et al., 2005, 2006; Onder et al., 2005; Buchman et al., 2007a,^2009^; Delmonico et al., 2007; Verghese et al., 2014; Ayers and Verghese, 2016; Callisaya et al., 2016). The field has not yet converged on the optimal metrics for assessing late-life motor impairment. Further work is also needed to identify motor or non-motor metrics that improve the specificity of motor testing for predicting distinct adverse health outcomes. Rapid advances in unobtrusive portable technologies have facilitated the ease for deploying instrumented gait testing outside of specialized gait labs. Recent work suggests that different combinations of digital mobility metrics may be differentially associated with distinct adverse health outcomes (von Coelln et al., 2019; Buchman et al., 2020a). So, adding digital mobility metrics to conventional motor metrics may lead to risk models that can identify subgroups of vulnerable older adults at risk for specific adverse health outcomes.

### Mixed-Brain Pathologies Account for a Minority of Motor Decline in Aging Adults

Evidence from brain imaging and post-mortem pathologic studies suggests that over many years aging brains accumulate diverse neurodegenerative and cerebrovascular disease (CVD) pathologies (Montine et al., 2012; Albers et al., 2015; White et al., 2016; Dodge et al., 2017). In very old adults, it is most common to observe multiple brain pathologies (Boyle et al., 2019; Yu et al., 2019b). In prior studies we found that the rate of late-life motor decline is associated with both Alzheimer’s disease (both amyloid beta and tau-tangles) and other as well as CVD brain pathologies (Buchman et al., 2013, 2014, 2015, 2020b,2021b). For example, a higher burden of mixed-brain pathologies is associated with a more rapid rate of motor decline quantified as parkinsonian signs is both adults with and without a clinical diagnosis of Parkinson’s disease (Boyle et al., 2013, 2017; Yu et al., 2015a; Wilson et al., 2016; Buchman et al., 2019b).

**FIGURE 1:**
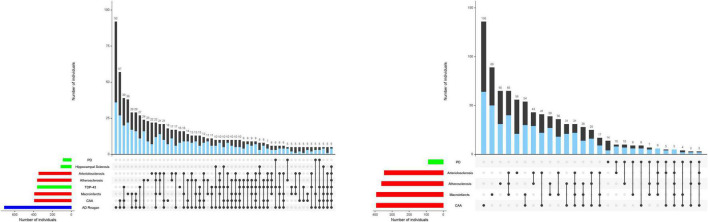
Combinations of brain pathologies associated with the rate of cognitive and motor decline in the same individuals. The bar charts below show the frequencies of individual brain pathologies indices collected in this study which were associated with either cognitive (left) and/or motor decline, based on 26 items from a modified Unified Parkinson’s Rating Scale (right) using two separate linear mixed effect models. One or more pathologies were observed in almost 95% of decedents. Connected black dots on the x-axis indicate the specific combination of brain pathology in five or more individuals. The second bar chart in the main panel show the frequencies of the brain pathology indices for persons with (blue) and without dementia (black) on the left and with (blue) or without parkinsonism (black) on the right ordered by their frequency. The height of each bar corresponds to the number of persons with each combination. AD = Alzheimer’s disease pathology; CAA = cerebral amyloid angiopathy. As illustrated in the figure, brain pathology indices frequently co-occur. More than 80% of older adults in these analyses showed combinations of two or more pathologies. Figure 1 is based on Boyle et al. (2018) and Buchman et al. (2021b).

The person-specific negative impact of a single pathology will depend on the combinations of brain pathologies accumulating in an individual’s aging brain (Boyle et al., 2018; Buchman et al., 2021b). Thus, the varied combinations of brain pathologies accumulating in aging brains may account not only for individual differences in motor and cognitive decline, but also the phenotypic heterogeneity of motor and cognitive decline for a single individual (Figure 1). Using novel analytic techniques, a recent study reported that accumulating brain pathologies are associated with not only the linear rate of motor decline, but may also explain in part the visit-to-visit variability in motor performance in older adults without overt neurologic diseases (Buchman et al., 2020b). Linear motor decline and the variability of motor decline may be associated with different pathologies. These findings highlight that the negative effects of brain pathologies may be complex and vary with different aspects of motor function. These associations highlight the necessity for further studies to elucidate the varied mechanisms that underlie the contributions of varied pathologies to the phenotypic heterogeneity of late-life motor impairment.

Imaging and post-mortem studies that have quantified postmortem indices of pathologies in older adults with motor testing prior to death have focused almost exclusively on the cerebrum. While the burden and various combinations of brain pathologies are related to motor decline, the overall variance of motor decline accounted for by brain pathologies is only 10–20% as compared to nearly 50% for cognitive decline (Boyle et al., 2013, 2019; Buchman et al., 2013, 2014, 2015, 2020b,2021b; Yu et al., 2019b). Brain pathologies may only account for a minority of the variance of motor decline since the instruments used to measure cognition may capture more aspects of cognitive function than those used to assess motor function. Alternatively, in contrast to cognition, motor pathways extend beyond the brain to peripheral muscle and most studies have only measured pathologies in the brain.

A handful of studies have collected pathologies from select regions outside of the brain in decedents with motor measure prior to death. Pathologies and degenerative changes outside of the brain account for additional variance of motor decline unexplained by brain pathologies alone (Buchman et al., 2012, 2017, 2018, 2019a). For example, microvascular pathologies accumulate throughout the spinal cord, but are not considered when evaluating older adults for potential cerebrovascular diseases (Buchman et al., 2017). Thus, to determine the full extent to which pathologies and degeneration contribute to motor decline in older adults it would be necessary to quantify degenerative changes in the entire motor pathway. To date, no studies have collected indices of pathologies and degeneration in the entire nervous system motor pathway including brain, brainstem, spinal cord, nerve and muscle, let alone the musculoskeletal system, in the same well-characterized older adults. Thus, the overall contribution of pathologies and degeneration in motor systems account for late-life motor decline is currently unknown.

### Resilience Genes and Proteins Account for Motor Decline Unexplained by Pathologies

Though nearly all aging brains show the accumulation of some degree of brain pathologies, the extent to which a unit of a brain pathology is associated with impaired cognitive or motor function has been observed to vary widely among older adults (Katzman et al., 1988; Sonnen et al., 2011; Stephan et al., 2012; White et al., 2016; Stern et al., 2019b,^2020^; Buchman et al., 2020b,c). We refer to this unexplained residual decline as resilience (Yu et al., 2015b; Boyle et al., 2021). While this catch all term almost assuredly includes some unmeasured pathologies, it also reflects the ability of the nervous system to be more or less tolerant of the measured pathologies. We have taken a similar approach to cognitive resilience thereby identifying numerous genes and proteins associated with slower or faster cognitive decline after accounting for the effects of nearly a dozen pathologies (Mostafavi et al., 2018; Yu et al., 2018, 2020). Similarly, the same burden of brain pathology may be related to more rapid motor decline in one adult with lower-than-average resilience and minimal motor decline in another individual with higher-than-average- resilience. This hypothesis has served to focus efforts to identify modifiable risk factors, lifestyles or molecular mechanisms that may provide motor resilience i.e., account for slower or faster motor decline that is independent of the negative effects of brain pathologies.

A wide variety of mechanisms may underlie resilience. In addition, to structural redundancies, the brain is plastic, actively responding to damage, behavior and past experiences. Resilience also includes both functional compensation through engagement of redundant neuronal populations and dynamic molecular resilience to maintain cellular homeostasis to counteract senescence. Resilience may be responsive to behavioral or life-style interventions (Zhou and Shine, 2003; Bennett et al., 2006; Boyle et al., 2012; Intlekofer and Cotman, 2013; Lim et al., 2013; Dawe et al., 2014, 2016; Wilson et al., 2015; Buchman et al., 2016c; Herring et al., 2016; Tapia-Rojas et al., 2016; Yu et al., 2016; Canli et al., 2018). For example, higher levels of physical activity may slow motor and cognitive decline (Buchman et al., 2019c; Oveisgharan et al., 2019). The biology underlying the resilience afforded by risk factors or lifestyles such as physical activity unrelated to brain pathologies are unknown and remain to be identified (Oveisgharan et al., 2020).

### Isolating Motor Resilience Is Crucial for Identifying Resilience Genes and Proteins

Structural (e.g., cytoskeleton, channel) and effector (e.g., signaling, enzymes) proteins are the primary physical bases of neural networks linking risk factors with motor decline. Neurodegenerative pathologies are associated with misfolded or abnormally activated proteins which drive the negative effects of accumulating brain pathologies on motor function. Other unidentified proteins, unrelated to the presence of brain pathologies, also contribute to motor decline. Motor resilience is defined as motor decline unrelated to brain pathologies. Thus, to elucidate the molecular mechanisms driving motor resilience it is necessary to isolate motor decline unrelated to brain pathologies from motor decline related to brain pathologies.

Little prior research has examined motor decline unexplained by brain pathologies because of the inherent challenges, including the need for longitudinal motor function over many years prior to death, and adequate measures of pathologies and resilience markers. Studies restricted to clinical and biomarker data can only account for some underlying pathologies (e.g., macroinfarcts, CSF and/or PET amyloid and tau). Other pathologies (e.g., microinfarcts, TDP-43) and resilience markers can only be studied in post-mortem brain tissue.

Genes and proteins which may contribute to motor resilience may be found in the widely distributed CNS tissues which support motor control systems. In prior work focusing on cognitive decline, we employed an analytic approach that regressed out the effects of diverse brain pathologies on cognitive decline to isolate residual cognitive decline i.e., cognitive decline unrelated to brain pathologies (Yu et al., 2015b). We then interrogated cognitive resilience in the dorsal lateral prefrontal cortex (residual decline) to discover a number of genes and proteins associated with cognitive resilience (Mostafavi et al., 2018; Yu et al., 2018, 2020). We used a similar approach to isolate motor decline unrelated to brain pathologies to identify genes and proteins associated with motor resilience (Buchman et al., 2021c).

**FIGURE 2:**
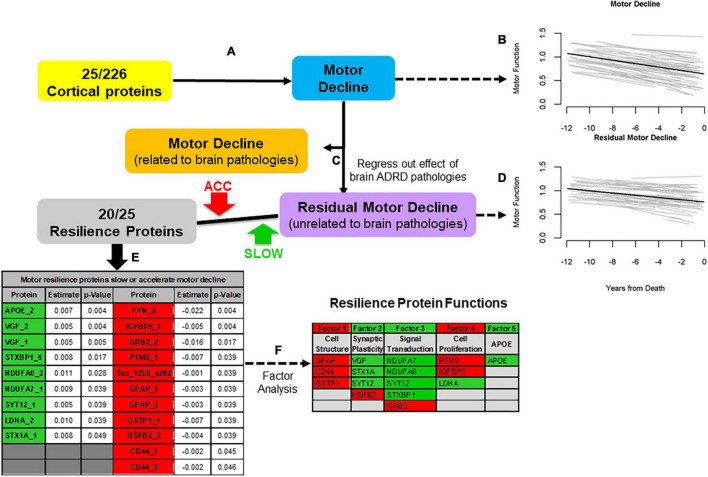
An approach to identify cortical proteins associated with motor resilience. We identified cortical proteins which were associated with motor decline **(A)**. We used linear-mixed effect model to examine the association between 226 proteins, measured in the dorsal lateral prefrontal cortex, with the rate of motor decline based on a summary measure of ten motor performances, controlling for age and sex. There were 25 proteins associated with motor decline after FDR correction. To illustrate the heterogeneity of motor decline, we show the trajectories of motor decline in a randomly selected group of 71 individuals included in these analyses. Trajectories of motor decline is based on repeated measures of motor testing prior to death. Each individual light line represents the estimated person-specific decline for an individual adult with the length of the line based on the number of years of follow-up. Bold black line represents average motor decline **(B)**. Motor decline can be partitioned in to two components. Some but not all of motor decline is explained by the negative effects of brain pathologies (orange box) and some is not explained by brain pathologies (purple box). Cortical proteins associated with motor decline not explained by brain pathologies may provide motor resilience. Therefore, we added terms for 10 indices of brain pathologies to the models of the 25 proteins associated with motor decline to regress out motor decline related to brain pathologies **(C)**. Trajectories of residual motor decline to capture the residual heterogeneity of motor decline after adding terms to the models **(A)** for ten indices of brain pathologies. Light lines show person-specific residual motor decline and bold black line represents average residual motor decline **(D)**. Five of 25 proteins were no longer associated with motor decline after adding terms for brain pathologies. Twenty of 25 proteins remained associated with motor decline not explained by brain pathologies after correction for FDR (**E**, Table). These 20 proteins may provide motor resilience to offset the deleterious motor effects of brain pathologies which commonly accumulate in aging brains. Higher levels of some proteins are associated with slower motor decline (Green) and higher levels of some proteins are associated with faster motor decline (Red). An exploratory factor analysis suggested that these twenty proteins clustered into five factors that share common physiologic functions (**F**, Table); Further details about the factors in this table are included in the Supplementary Table 3 in Buchman et al. (2021c). [Figure based on Buchman et al. (2021c)].

We leveraged data from older autopsied decedents from one of two cohort studies with longitudinal motor function over many years prior to death with adequate measures of ten post-mortem brain pathologies in whom cortical proteins that might provide motor resilience were measured. We quantified 226 proteotypic peptides in the dorsolateral prefrontal cortex. First, we identified 25 of 226 proteins that were related to motor decline based on a summary measure of ten conventional motor performances prior to death (Figures 2A,B). Twenty of 25 peptides (Figure 2E) remained associated with motor decline in models controlling for ten brain pathologies that isolated motor resilience (Figures 2C,D). Higher levels of nine peptides (Figure 2E, green) were related to slower motor decline and higher levels of eleven peptides were related to faster decline (Figure 2E, Red). These proteins may share common physiologic functions (Figure 2F).

We then aggregated the expression levels of these twenty motor resilience peptides to yield a person-specific motor resilience score to test whether an aggregated resilience score might identify adults with higher or lower than average motor resilience (Buchman et al., 2021c). Supporting this notion, we found that a higher motor resilience score was associated with slower motor decline, less severe parkinsonism and disabilities proximate to death. These findings suggest that all individuals in these analyses manifest some degree of motor resilience with some having higher than average and some having lower than average resilience.

Building on these findings, we used the same approach described above to examine motor decline to identify cortical resilience proteins related to two additional aging phenotypes cognitive decline and progressive parkinsonism in the same individuals (Zammit et al., 2022). Cognitive decline was based on a summary score for global cognition that summarized seventeen cognitive tests. Progressive parkinsonism was based on a composite score that summarized parkinsonian signs based on assessment of twenty-six items from the unified Parkinson rating scale. We compared the lists of proteins obtained from the complementary analyses of these three aging phenotypes. About 70% of the proteins provided resilience for a distinct phenotype i.e., motor decline, cognitive decline or progressive parkinsonism and about 30% of the proteins provided resilience for more than one of the three aging phenotypes that were examined. This suggests that some cortical proteins may provide resilience for distinct phenotypes and a minority may provide resilience for multiple aging phenotypes. It is not surprising that some cortical proteins are pleotropic and may have more than one function. In fact, we have also shown that some resilience proteins may also reduce the negative effects of brain pathologies *via* multiple pathways. Some pathways are related and some pathways are unrelated to AD/ADRD pathological traits (Yu et al., 2020; Zammit et al., 2022).

### Implications and Future Directions

Progressive loss of motor function in old age results from a complex interaction between the accumulation of mixed-pathologies and degenerative changes in motor tissues within and outside CNS that manifest more or less resilience to these changes. Despite advances in our understanding of the role of brain pathologies to the phenotypic heterogeneity of motor decline, treatments are lacking for nearly all currently recognized neuropathologies. Moreover, aging brains show combinations of diverse ADRD pathologies and degeneration, so even successful treatments for an individual pathology are likely to have only a small effect on overall motor impairment. Also, efforts to advance treatments are complicated by the limited data about the extent to which degenerative changes in motor tissues outside of the brain contribute to motor decline. These knowledge gaps underscore the potential advantages of interventions targeting neural motor resilience proteins that may offset the negative effects of multiple pathologies.

Recent work focusing on the identification of genes and proteins underlying resilience suggests a more expansive concept of resilience. Some limit the concept of resilience to factors which have a unidirectional beneficial effect on an individual’s ability to maintain function despite accumulating pathology (Esiri and Chance, 2012; Barulli and Stern, 2013; Stern et al., 2019a). Thus, if this factor is absent there is no resilience. This approach treats resilience and vulnerability in older adults as independent concepts. The work discussed above identified some proteins related to slower and other proteins related to faster motor or cognitive decline. This suggests that resilience like many other conventional risk factors is a continuum. Hence, all living brains have some degree of resilience i.e., the balance between many proteins, some increase and some decrease brain resilience. This supports a conceptualization of resilience in old age as either more or less than the average reference group, an approach widely used in analytic epidemiologic studies of continuous exposures, e.g., more or less physically active, higher or lower body mass index. Explicating the biology of resilience may inform not only on efforts to promote resilience, but may provide new approaches to reverse vulnerability in aging adults.

Initial efforts to aggregate the many proteins driving resilience into a summary score highlight how these efforts may lead to clinical tools for risk stratification of vulnerable older adults and improve the homogeneity of clinical trials. The genes or proteins discovered using this approach will require further mechanistic studies and validation studies in model organisms or human cell modeling before they can be translated into the clinical domain. Unbiased genome wide or proteome wide studies are needed to more fully capture molecular mechanisms that may provide neural motor resilience. To date human studies of neural resilience have been limited to the brain. It will also be important to extend a similar approach to regions outside the brain to discover additional motor resilience mechanisms and the extent to which these mechanisms are conserved within distributed motor pathways. Finally, isolating resilience and applying genomics to investigate resilience also has potential to yield high value therapeutic targets for further drug discovery that can lead to novel targeted therapies that prevent late-life motor impairment despite the presence of untreatable pathologies and degeneration.

## Data Availability Statement

This perspective discussed data from prior publications. These data can be obtained from the Rush Alzheimer’s Disease Center (RADC) repository at http://www.radc.rush.edu.

## Ethics Statement

The studies involving human participants were reviewed and approved by Institutional Review Board of Rush University Medical Center. The patients/participants provided their written informed consent to participate in these studies.

## Author Contributions

AB and DB contributed to conception and design of the manuscript, manuscript revision, read, and approved the submitted version. AB wrote the first draft of the manuscript. Both authors contributed to the article and approved the submitted version.

## Conflict of Interest

The authors declare that the research was conducted in the absence of any commercial or financial relationships that could be construed as a potential conflict of interest.

## Publisher’s Note

All claims expressed in this article are solely those of the authors and do not necessarily represent those of their affiliated organizations, or those of the publisher, the editors and the reviewers. Any product that may be evaluated in this article, or claim that may be made by its manufacturer, is not guaranteed or endorsed by the publisher.
